# Gemella endocarditis

**DOI:** 10.1016/j.idcr.2022.e01597

**Published:** 2022-08-08

**Authors:** Hussein Rabah, Khalil El Gharib, Marc Assaad, Ali Kassem, Neville Mobarakai

**Affiliations:** a Department of Medicine, Staten Island University Hospital, Staten Island, NY 10305, USA; b Department of Infectious Disease, Staten Island University Hospital, Staten Island, NY 10305, USA

**Keywords:** Infective endocarditis, Valvular disease, Mitral valve, Echocardiogram, Blood culture, Antibiotic therapy

## Abstract

We herein present the case presenting to our facility complaining of a fever of two months duration, who underwent dental procedure. Patient was diagnosed with infective endocarditis secondary to an uncommon bacteria: *Gemella haemolysans*. Patient was found to have concomitant severe mitral valve regurgitation. Our patient did not have any comorbidity or risk factor beside his dental procedure. Our patient received intravenous antibiotic therapy for six weeks and was scheduled for mitral valve replacement.

## Introduction

Infective endocarditis (IE) is a life-threatening disease resulting from bacterial or fungal infection of the endocardium, the heart's inner surface. It most often affects the heart valves, particularly the prosthetic ones, and is associated with a 30-day mortality rate of 10–30 % [1].

Several risk factors predispose to the development of IE, including valvular disease and prosthetic valves, congenital heart disease, previous infective endocarditis, intravascular devices, cardiac implantable devices, in addition to injection drug use [2], [3], [4].

The pathophysiology of infective endocarditis results from complex interactions of several independent factors. The human endocardium is resistant to bacterial colonization [5]; thus, the development of endocarditis requires damage to the cardiac endothelium in order to facilitate bacterial attachment. Such alterations in the inner cardiac surface usually result from turbulent blood flow, endocardial impairment due to primary or secondary valvular disease, or injury due to foreign particles resulting from intravenous drug use. The injury facilitates the formation of fibrin-platelet clots on the surface of the traumatized epithelium, leading to "nonbacterial thrombotic endocarditis" (NBTE) [5], [6]. This adhesion promotes the colonization of bacteria in the bloodstream to the affected area of the endocardium, further deposition of fibrin and platelets, the proliferation of the bacteria, and subsequent vegetation formation [7].

The diagnosis of IE might be challenging as the presentation is usually atypical; thus, the modified Duke's criteria aid in diagnosing IE based on clinical, echocardiographic, microbiological, and immunological criteria [8].

*Staphylococcus aureus* is the leading microorganism causing IE. Other bacteria *include Viridans group streptococci* (VGS*), coagulase-negative staphylococci* (CoNS), and *Enterococcus spp* [9]. This report presents a case of mitral valve IE caused by *Gemella haemolysans* in order to further understand this pathogen as literature reporting this microorganism is scarce. Patient’s consent for publication was obtained.

## Case presentation

A 56-year-old male with no past medical history presented for 2-month history of intermittent low-grade fever. He underwent a root canal dental procedure about 9 weeks before his presentation without receiving any antibiotic prophylaxis. He denied any other complaints.

On physical examination, the patient was hemodynamically stable and afebrile. On cardiac auscultation, he had a 5/6 apical holosystolic murmur radiating to his left axilla. His skin was normal, without rashes or nodules, and his ophthalmoscopic examination was negative.

**Fig. 1:**
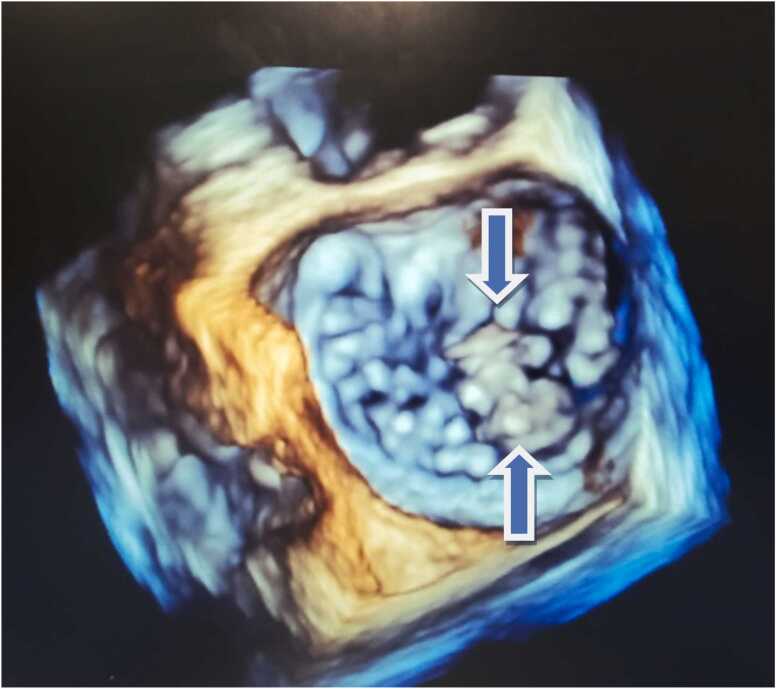
Three dimensional Echocardiography showing the mitral vegetation.

**Fig. 2:**
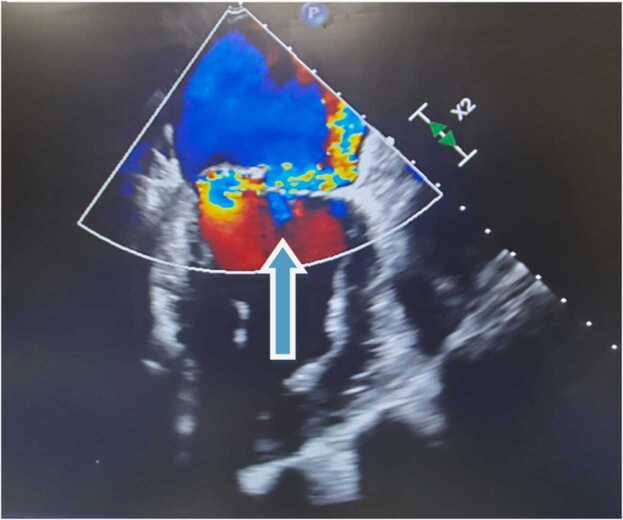
Transesophageal Echocardiogram showing severe mitral regurgitation on color doppler ultrasound.

His blood work showed a white cell count of 7.35 k/µL, hemoglobin of 11.4 g/dL, and a creatinine level of 0.8 mg/dL. His urine analysis was negative for microscopic hematuria. A bedside transthoracic echocardiogram was concerning for possible mitral valve vegetation. Transesophageal echocardiography demonstrated posterior mitral leaflet vegetation consistent with IE with moderate to severe mitral regurgitation (Fig. 1, Fig. 2). After blood cultures were drawn, the patient was started on vancomycin and ceftriaxone.

Two sets of blood cultures grew gram-positive cocci in pairs identified later as *G. haemolysans*. The multiplex polymerase chain reaction PCR assay was perfomed which failed to identify resistant gene targets. Vancomycin was stopped, and ceftriaxone monotherapy was continued. The bacteremia resolved 2 days after antibiotic therapy initiation. In the meantime, susceptibilities were perfomed which confirmed later on the multisensitive nature of this bacteria. Due to severe mitral valve regurgitation, the Cardiothoracic surgery team was consulted to evaluate mitral valve replacement after adequately treating the endocarditis. The patient was then discharged on antibiotic therapy via a peripheral inserted central catheter (PICC line) for 6 weeks and scheduled for mitral valve replacement.

The patient had no embolic events during their admission, and his hospital stay was uncomplicated.

## Discussion

*Gemella* was first described in 1917 as a gram-positive coccal, facultative anaerobic microorganism, and it was initially classified as Neisseriaceae [10], [11]. The identification of *Gemella* isolates represents a challenge to laboratories. These bacteria are easily decolorized during gram stains and mistakenly categorized as gram-negative organisms. Sometimes, *Gemella haemolysans* can be misidentified as *strep viridans* or remain unidentified [12]. Therefore, accurate diagnosis of such an organism is crucial to promptly manage the patients.

*G. haemolysans* colonizes the oral cavity, upper respiratory, and gastrointestinal tracts. Although infections caused by this microorganism are very rare, it can cause severe localized and systemic diseases, including central nervous system infections [13], [14], osteomyelitis [15], and endocarditis [16]. Generally, infections due to *Gemella* are associated with an underlying medical condition or procedures [16]. The patient presented in this report had no health problems, but he underwent a dental procedure 9 weeks prior to his presentation. Although it is very likely that the endocarditis in this case have resulted from the dental procedure, however bacterial seeding from the oral cavity might have resulted from any dental manipulation such as toothbrushing.

*Gemella spp* can adhere to the oral cavity surfaces using adherent proteins equivalent to those of *viridans streptococci*, thus providing the perfect media for functional and structural demands and a route for systemic invasion after injury and trauma [17]. Furthermore, *G. haemolysans* and *strep mitis* share structural features of IgA proteases, including zinc-dependent metalloproteases. Those proteases aid in human IgA destruction [18], which might explain why dental manipulations precede most endocarditis caused by *Gemella isolates*.

Several proteins facilitate the pathogen binding to the host extracellular matrix (ECM). The ECM is vital for the proliferation, differentiation, and survival of the invading organism. *Gemella* isolates contain adhesive proteins with Fibronectin binding protein A similar to that of *streptococci* endocarditis isolates, suggesting a significant role in this disease [17].

In addition, *G. haemolysans* and *G. morbillorum carry exclusive surface* lipoproteins PsaA responsible for adhesion, immune system evasion, and nutrient scavenging [17]. Those complex factors may favor the bacterial colonization of the EMC of the damaged endocardium leading to bacterial proliferation and endocarditis.

Gemella isolates are usually sensitive to beta-lactams and vancomycin [19]. One treatment choice reported in early literature describing Gemella endocarditis is penicillin and Gentamicin [20], and this explains why Gentamicin is usually added as an antimicrobial therapy. Our patient was treated empirically with ceftriaxone and vancomycin, then switched to ceftriaxone monotherapy after blood culture results. Bacteremia resolved after 2 days of antibiotic initiation, and he was treated for a total of 6 weeks with no complications. This shows that B lactam monotherapy is effective in treating Gemella endocarditis.

## Conclusion

Gemella Haemolysans is a rare cause of IE, and its diagnosis represents a challenge for clinicians. The primary source of Gemella is the oral cavity. Early recognition of this pathogen results in prompt treatment and avoidance of complications. Furthermore, Ceftriaxone monotherapy is effective in controlling the infection.

## CRediT authorship contribution statement

All authors have made contributions to writing this case report. Neville Mobarakai have supervised and edited the paper. Hussein Rabah is responsible for the concept. Hussein Rabah, Khalil El Gharib, Marc Assaad and Ali Kassem drafted the manuscript. Marc Assaad and Ali Kassem are responsible for collecting the resources.

## Funding

This paper received no grants from any funding campaign.

## Ethical approval

Not applicable.

## Consent

Written informed consent was obtained from the patient for publication of this case report and accompanying images. A copy of the written consent is available for review by the Editor-in-Chief of this journal on request.

## Competing interests

The authors declare that there is no conflict of interest.

## References

[1] Que Y.A., Moreillon P. (2011). Infective endocarditis. Nat Rev Cardiol.

[2] Richey R., Wray D., Stokes T., Guideline Development Group (2008). Prophylaxis against infective endocarditis: summary of NICE guidance. BMJ.

[3] Martín-Dávila P., Fortún J., Navas E., Cobo J., Jiménez-Mena M., Moya J.L. (2005). Nosocomial endocarditis in a tertiary hospital: an increasing trend in native valve cases. Chest.

[4] Chahoud J., Sharif Yakan A., Saad H., Kanj S.S. (2016). Right-sided infective endocarditis and pulmonary infiltrates: an update. Cardiol Rev.

[5] Durack D.T., Beeson P.B., Petersdorf R.G. (1973). Experimental bacterial endocarditis. 3. Production and progress of the disease in rabbits. Br J Exp Pathol.

[6] Gross L., Friedberg C.K. (1936). Nonbacterial thrombotic endocarditis: classification and general description. Arch Intern Med.

[7] McGowan D.A., Gillett R. (1980). Scanning electron microscopic observations of the surface of the initial lesion in experimental streptococcal endocarditis in the rabbit. Br J Exp Pathol.

[8] Li J.S., Sexton D.J., Mick N., Nettles R., Fowler V.G., Ryan T. (2000). Proposed modifications to the Duke criteria for the diagnosis of infective endocarditis. Clin Infect Dis.

[9] Vogkou C.T., Vlachogiannis N.I., Palaiodimos L., Kousoulis A.A. (2016). The causative agents in infective endocarditis: a systematic review comprising 33,214 cases. Eur J Clin Microbiol Infect Dis.

[10] Facklam R., Elliott J.A. (1995). Identification, classification, and clinical relevance of catalase-negative, gram-positive cocci, excluding the streptococci and enterococci. Clin Microbiol Rev.

[11] Thjötta T., Böe J. (1938). Neisseria hemolysans. A hemolytie species of Neisseria trevisan. Acta Pathol Microbiol Scand.

[12] Ruoff K.L. (1990). Gemella: a tale of two species (and five genera). Clin Microbiol Newsl.

[13] Anil M., Ozkalay N., Helvaci M., Agus N., Guler O., Dikerler A. (2007). Meningitis due to Gemella haemolysans in a pediatric case. J Clin Microbiol.

[14] Lee M.R., Lee S.O., Kim S.Y., Yang S.M., Seo Y.H., Cho Y.K. (2004). Brain abscess due to Gemella haemolysans. J Clin Microbiol.

[15] Fangous M.S., Hémon F., Graf P., Samier-Guérin A., Alavi Z., Le Bars H. (2016). Bone infections caused by Gemella haemolysans. Med Mal Infect.

[16] Kariyanna PT, Sutarjono B, Ellanti NP, Jayarangaiah A, Jayarangaiah A, Chandrakumar HP, et al. Risk factors and patient profile of infective endocarditis due to Gemella spp. Am J Med Case Rep, Vol. 9(no. 2); 2021, p. 103–15. 〈 10.12691/ajmcr-9-2-4〉. [Epub 2020 Dec 13. PMID: 33585676; PMCID: PMC7877815].

[17] García López E., Martín-Galiano A.J. (2020). The versatility of opportunistic infections caused by *Gemella* isolates is supported by the carriage of virulence factors from multiple origins. Front Microbiol.

[18] Takenouchi-Ohkubo N., Mortensen L.M., Drasbek K.R., Kilian M., Poulsen K. (2006). Horizontal transfer of the immunoglobulin A1 protease gene (iga) from Streptococcus to Gemella haemolysans. Microbiology.

[19] Buu-Hoï A., Sapoetra A., Branger C., Acar J.F. (1982). Antimicrobial susceptibility of Gemella haemolysans isolated from patients with subacute endocarditis. Eur J Clin Microbiol.

[20] Jonathan Baghdadi, Kelesidis Theodoros, Humphries Romney (2015). In vitro susceptibility of Gemella species from clinical isolates. Open Forum Infect Dis.

